# Evaluation of a new set of recombinant antigens for the serological diagnosis of human and canine visceral leishmaniasis

**DOI:** 10.1371/journal.pone.0184867

**Published:** 2017-09-28

**Authors:** Franklin B. Magalhães, Artur L. Castro Neto, Marilia B. Nascimento, Wagner J. T. Santos, Zulma M. Medeiros, Adelino S. Lima Neto, Dorcas L. Costa, Carlos H. N. Costa, Washington L. C. dos Santos, Lain C. Pontes de Carvalho, Geraldo G. S. Oliveira, Osvaldo P. de Melo Neto

**Affiliations:** 1 Associação Caruaruense de Ensino Superior e Técnico, Caruaru, Pernambuco, Brazil; 2 Centro de Pesquisas Aggeu Magalhães, Fundação Oswaldo Cruz (Fiocruz-Pernambuco), Recife, Pernambuco, Brazil; 3 Instituto de Doenças Tropicais Natan Portella (IDTNP), Teresina, Piaui, Brazil; 4 Centro de Pesquisas Gonçalo Moniz, Fundação Oswaldo Cruz (Fiocruz-Bahia), Salvador, Bahia, Brazil; Academic Medical Centre, NETHERLANDS

## Abstract

Current strategies for the control of zoonotic visceral leishmaniasis (VL) rely on its efficient diagnosis in both human and canine hosts. The most promising and cost effective approach is based on serologic assays with recombinant proteins. However, no single antigen has been found so far which can be effectively used to detect the disease in both dogs and humans. In previous works, we identified *Leishmania infantum* antigens with potential for the serodiagnosis of VL. Here, we aimed to expand the panel of the available antigens for VL diagnosis through another screening of a genomic expression library. Seven different protein-coding gene fragments were identified, five of which encoding proteins which have not been previously studied in *Leishmania* and rich in repetitive motifs. Poly-histidine tagged polypeptides were generated from six genes and evaluated for their potential for diagnosis of VL by ELISA (Enzyme Linked ImmunoSorbent Assay) with sera from infected humans and dogs. None of those was valid for the detection of human VL (26–52% sensitivity) although their performance was increased in the canine sera (48–91% sensitivity), with one polypeptide useful for the diagnosis of canine leishmaniasis. Next, we assayed a mixture of three antigens, found to be best for human or canine VL, among 13 identified through different screenings. This “Mix” resulted in similar levels of sensitivity for both human (84%) and canine (88%) sera. With improvements, this validates the use of multiple proteins, including antigens identified here, as components of a single system for the diagnosis of both forms of leishmaniasis.

## Introduction

Visceral leishmaniasis (VL) is a systemic and chronic disease caused by an intracellular protozoan parasite of the genus *Leishmania* and transmitted via sand flies. It is usually characterized by long duration fever, weight loss, weakness, lethargy, splenomegaly, and pancytopenia, among other manifestations [1–4]. In epidemiological terms, VL is classified into anthroponotic and zoonotic types, with the predominant causal agent for the anthroponotic type being *Leishmania donovani* and for the zoonotic type being *Leishmania infantum*, previously known as *Leishmania chagasi* in Latin America [1,5]. In Brazil, this disease is a public health problem with high transmission and case fatality rates [6,7].

The domestic dog is considered to be the main reservoir of *L*. *infantum*, whereas the possibility remains for wild animals such as foxes and marsupials acting as sylvatic reservoirs [8,9]. In general, the clinical signs of the canine leishmaniasis and its diagnosis in sick dogs are relatively clear, although the same cannot be said of animals displaying few signs or those which are apparently healthy [10]. Afflicted animals can display signs of the disease soon after infection or remain subclinically infected for significant lengths of time, but both are infectious to the sandfly vector [11].

Detection of the parasite through direct microscopic visualization or culturing from biological samples derived from infected individual is the gold standard of VL diagnosis but this is not practical on a large scale. Early serological methods used for VL serodiagnosis (e.g. the direct agglutination test or DAT and immunofluorescence-based tests), relied on the use of whole extracts and fixed parasites. Diagnostic tests based on the detection of the parasite DNA by PCR have also been implemented and they have the advantage of being able to discriminate an active infection from those in which the parasite has been eliminated. All, however, have cost related issues, require a more complex infrastructure and cannot be implemented in the field [11–15].

An alternative for improving the serological techniques used for VL diagnosis is the use of specific molecules that are readily recognized by most sera from infected individuals. For this purpose several recombinant antigens have been tested and the most promising antigen for VL diagnosis so far, is the rK39 [15,16]. This antigen is based on a 39 amino acid repeat derived from a *L*. *infantum* kinesin-related polypeptide [17] and early studies indicated its potential for the diagnosis of human VL and the evaluation of its progression [18]. Extensive field tests have been carried out since then using this antigen in different formats and an rK39 strip test, readily applicable in the field, has been shown to be a sensitive and a reliable indicator of VL in human patients. In a meta-analysis study evaluating the data from independent studies using this rK39 strip test, the results were overall seen to be quite uniform with very high sensitivity and specificity [19]. In Eastern Africa, however, its performance was seen to be inferior to what was observed elsewhere [16–21] and in general it was seen to be positive in a significant proportion of healthy individuals from endemic regions and for extended periods after cure of the disease [14,22]. Another relevant aspect is that tests made in the field focusing on the diagnosis of VL in dogs did not show equivalent results to those seen with human samples and suggest that a rK39-based test may not be suitable for the identification of infected dogs on its own [23–25].

The humoral immune responses generated during an infection by *L*. *infantum* in infected dogs and humans are quite distinct and the antigens most suitable for the diagnosis of VL in humans are not necessarily valid to use with canine samples [26]. In a previous study our group identified and evaluated five antigens obtained through the screening of a *L*. *infantum* cDNA library for their potential for the diagnosis of both human and canine VL. Although some of the antigens displayed high sensitivity and specificity to one or the other species, none of them were effective for the serodiagnosis in both humans and dogs [23]. Within this context, we aimed at expanding the panel of available antigens for the diagnosis of VL through the evaluation of newly selected antigens, as well as a mixture of some of the best among those evaluated, for the diagnosis of both canine and human forms of the disease.

## Materials and methods

### Parasites

*Leishmania infantum* (MHOM/BR2000/Merivaldo2, 2240) promastigotes were maintained at 26°C in modified LIT medium, pH 7.2 [0.2% sucrose (w/v), 0.36% liver broth (w/v), 0.1% tryptose (w/v), 0.002% haemin (w/v)], or Schneider medium, pH 7.2 (Sigma-Aldrich), containing 10% fetal bovine serum, ampicilin (10 U/ml) and streptomycin (10 μg/ml). Parasites were kept on log phase growth by passaging to new culture media every 3 to 4 days. Total parasite lysate (LAg) was obtained by sonication of log-phase parasites. The protein content of the lysate was quantified by the Bradford method.

### Sera

The human sera panel was composed of: 50 clinically and parasitologically diagnosed VL patients obtained from an endemic area in Piaui State, Brazil, independently of sero-reactivity; 50 negative sera samples obtained from healthy individuals of various ages from Pernambuco State, also endemic for VL; 26 parasitologically confirmed cutaneous leishmaniasis patients; 40 serologically confirmed patients with chronic Chagas’ disease. The canine serum samples were obtained from: 46 dogs with parasitologically confirmed VL, from the endemic area of Jequié (Bahia state, Brazil); 15 healthy young dogs of various ages and breeds from non-endemic areas; and sera from 31 dogs afflicted with other infectious diseases (4 with babesiosis, 20 with erhlichiosis, and 7 with demodicosis) were also used.

### Ethical approval

All dogs were handled in agreement with the Oswaldo Cruz Foundation guidelines for experimentation on animals and the collection of the sera used in this study was approved by the ethics committee for the use of animals in research (CPqGM-FIOCRUZ, Ceua, license N.040/2005). All human sera were collected after approval of their use by the appropriate ethics committees, as follows: use of the sera from VL patients was approved by the ethics committee from the Federal University of Piaui (0116/2005); the negative control sera were included in the study approved by the ethics committee of the Brazilian Ministry of Health (25000.119007/2002-03); sera from patients with cutaneous leishmaniasis were included in the project CAEE 0014.0.095.000–05, approved by the ethics committee from CPqAM-FIOCRUZ (03/08/2008); and the samples from chagasic patients used in this study were obtained from the serum bank at the Reference Laboratory for Chagas Disease at CPqAM-FIOCRUZ. Written informed consent was obtained from all adults or legal guardians of children before blood was collected.

### *Leishmania infantum* genomic library and serological screening

A *L*. *infantum* genomic library was constructed with reagents from the Stratagene Corporation (La Jolla, USA) using total *L*. *infantum* DNA partially digested with Tsp5091 and ligated into the λ-ZAP Express bacteriophage, previously digested with EcoRI. Library amplification and screening was carried out as previously described [27], using a pool of six sera from patients with VL, at a 1:1000 dilution, to screen approximately 30.000 clones. The inserts were sequenced and compared with sequences from the *L*. *infantum* and *Leishmania major* genomes available at the TriTrypDB (http://tritrypdb.org/tritrypdb/) and GeneDB (www.genedb.org) databases. Sequences obtained from the ends of each insert were then used for BLAST searches against genomic nucleotide sequences of *L*. *infantum* available at TriTrypDB. For the 5' ends of nearly all selected clones (except Lci9), sequences identical in nearly 100% of the nucleotides were found within regions predicted as protein coding regions whereas the corresponding 3' ends matched nearby sequences within the same open reading frame or within the neighboring intergenic regions. For Lci9, not found within the available *L*. *infantum* databases, the sequence for the whole 2.4 kb insert was generated by direct sequencing. The final sequence has been deposited to GenBank and received the accession number **KX018626**.

### Subcloning strategies

For recombinant protein expression, a distinct strategy was used for each insert, depending on the occurrence of internal restriction sites compatible with the subcloning strategy into the expression plasmids of the pRSET series (A, B or C—Invitrogen), as follows: the Lci6 insert (3.8 Kb) was recovered after digestion with BamHI/KpnI and the insert ligated into the same sites of pRSET C; Lci7 (2.4 Kb) was recovered using BamHI/SalI and the insert was ligated into the BamHI/XhoI sites of the pRSET B; Lci9 (2.4 Kb) was recovered with BamHI/XhoI and ligated into the same sites of pRSET B; Lci10 (0.9 Kb) was also recovered with BamHI/XhoI but the insert was ligated into the same sites of pRSET C; Lci11 (1.9 Kb) was first recovered using the enzymes BamHI/NotI and subcloned into the same sites of the vector pET21a (Novagen), followed by a second subcloning event where the Lci11 fragment was recovered from the resulting plasmid using BamHI/XhoI and the insert ligated into the same sites of pRSET A; Lci12 (2.8 Kb) was subcloned in pRSET B after digestion with the enzymes BamHI/PstI. In all cases, the resulting constructs encode for fusion proteins containing a common 32 amino acid segment at their N-terminuses, derived from the pRSET vector and which includes a polyhistidine tract (MRGSHHHHHHGMASMTGGQQMGRDLYDDDDKD) plus, eventually, a few further amino acids encoded by the vector’s multiple cloning sites immediately before and after the antigen coding segment. For Lci13 it was generated after two sets of subcloning reactions, where first a 1 Kb PstI fragment from the previously described Lc2.2 clone [28] was subcloned into the PstI site of the pTZ18R vector, with its 5’ end facing the vector´s T7 promoter. For the second subcloning reaction, the insert was recovered from the pTZ18R vector through digestion with BamH I/Hind III and subcloned into the same sites of pRSET A. For Lci1, the plasmid used for its expression has been described before [23].

### Expression and purification of recombinant proteins

For the expression of His-tagged recombinant proteins, *Escherichia coli* BL21(DE3) pLysS (Invitrogen) bacteria were transformed with the pRSET derived plasmids, grown in LB medium and expression induced by IPTG. Induced cells were harvested, resuspended in 0.15 M phosphate buffered saline, pH 7.2 (PBS) and lysed by sonication. Protein purification was performed with Ni-NTA Agarose (Qiagen). Protein products were analysed by 15% polyacrylamide gel electrophoresis in the presence of sodium dodecyl sulfate (SDS-PAGE), followed by staining of the proteins with Coomassie blue R-250. For estimation of the recombinant proteins concentrations, the densities of their stained bands in Coomassie blue stained gels were compared with those of known concentrations of bovine serum albumin (BSA).

### ELISA

The ELISA assays were essentially carried out as previously described at [23]. Briefly, ~400 ηg of the individual recombinant proteins or mixes of three proteins consisting of 300 ηg of each protein were added to each well of ELISA plates. The wells were then incubated with the selected sera at a dilution of 1:200 (canine sera) or 1:900 (human sera), followed by incubation with the secondary antibody, namely peroxidase-conjugated goat anti-dog IgG (1:1200) or anti-human IgG (1:10000), depending on the tested sera. For the rK39 ELISA assays, the commercial recombinant rK39 antigen was purchased from Rekom Biotech (Granada, Spain) and the assays were carried out following the manufacturer’s specifications.

### Statistical analysis

The cutoff values for the ELISAs were defined as means of results obtained with serum samples from 50 healthy donors plus three standard deviations. The ROC curves and the sensitivity and specificity values were generated with the Medcalc Software version 15.8. The graphs were generated by the GraphPad Prism 3.

## Results

### Serological screening and identification of novel *Leishmania infantum* antigens

A total of 60 positive clones from a genomic *L*. *infantum* expression library were identified after an immunoscreening with a pool of six sera from Brazilian VL patients. Inserts from 50 clones were sequenced and seven different protein-coding gene fragments were identified and their protein products named as Lci6, Lci7, Lci8, Lci9, Lci10, Lci11, and Lci12, to avoid confusion with the five antigens previously described by us from *L*. *infantum* [23]. Thirty-three clones were found to contain fragments of the Lci6 gene, three encoded Lci7, two encoded Lci8 and the remaining genes (encoding Lci9, Lci10, Lci11 and Lci12) were represented by one clone each. When compared with *L*. *infantum* and *L*. *major* sequences, five of the identified antigens (Lci6, Lci8, Lci9, Lci10 and Lci12) are either annotated as hypothetical or have not been properly studied in *Leishmania*. Lci11 has been previously described from *Leishmania amazonensis* as a phosphoprotein which binds specifically to a homologue of the translation initiation factor eIF4E, named as Leish4E-IP (for 4E interacting protein) [29]. This is a hydrophilic protein conserved in *L*. *infantum* and *L*. *major* but with limited conservation in *Trypanosoma* spp and which is very rich in the amino acids proline, glutamine, alanine and serine. The seventh polypeptide, Lci7, is the *L*. *infantum* orthologue of the stress-inducible protein sti1, originally described in *L*. *major* [30]. Table 1 lists the *L*. *infantum* accession numbers from TriTrypDB for the genes encoding most of the identified proteins. For Lci9, its gene hasn’t been properly annotated within the *L*. *infantum* genomic sequences, although sequences resembling parts of this gene can be found split within two distinct segments of chromosome 28, suggesting an assemblage error perhaps due to the shotgun nature of the sequencing of this genome [31]. An orthologue for the Lci9 gene is clearly identifiable in *L*. *major*, however, and its accession number is also listed in Table 1.

**Table 1:** TriTrypDb accession numbers for the newly identified antigenic proteins.

Protein	Accession number in TriTrypDB
**Lci6**	LinJ.26.1950
**Lci7**	LinJ.08.1020
**Lci8**	LinJ.32.2420
**Lci9**	LmjF.28.3010
**Lci10**	LinJ.34.2360
**Lci11**	LinJ.35.4030
**Lci12**	LinJ.29.0110

### Sequence analysis of the novel *L*. *infantum* antigens

**Fig 1:**
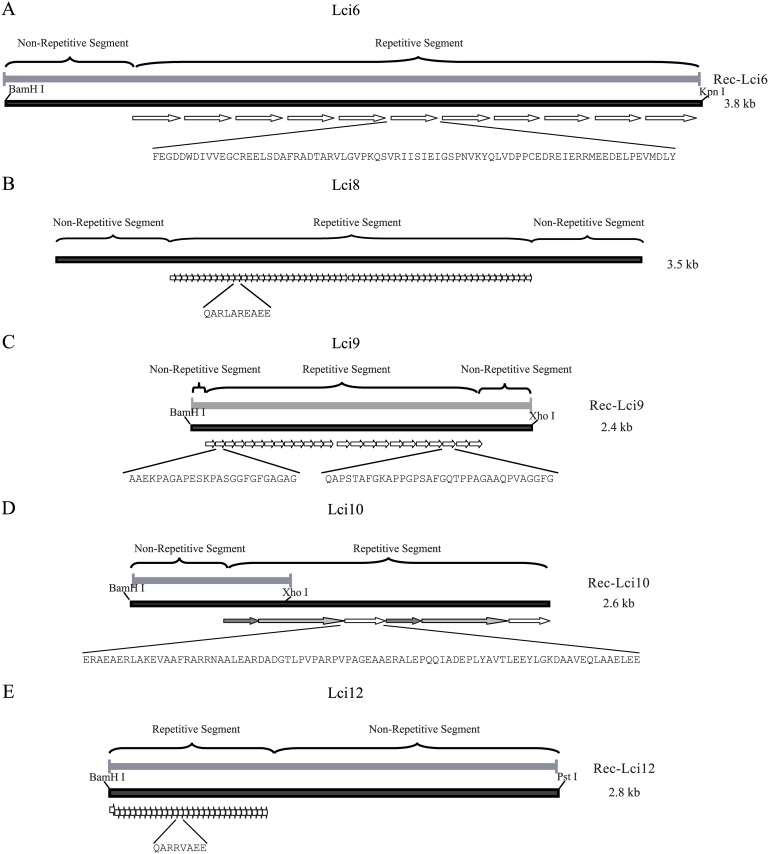
Schematic representation of the various gene fragments and corresponding deduced recombinant proteins evaluated in this study. The maps were derived from the sequences produced after direct sequencing or from the coding genomic sequences available at TriTrypDb.

All five novel *Leishmania* antigens identified in the *L*. *infantum* screening (Lci6, Lci8, Lci9, Lci10 and Lci12) have in common the presence of tracts of *in tandem* repetitive motifs (Fig 1). Most of these (Lci6, Lci8, Lci10 and Lci12) are predicted to be large proteins. The length of their repeats varies significantly, from only 8 (Lci12) to more than 50 (Lci6 and Lci10) amino acid residues, but little similarity in sequence between the repeats is observed, with the exception of those from Lci8 and Lci12, which seem to be related. Lci6 is composed by a non-repetitive N-terminal region (≈450 residues long), followed by 21 non-identical repeats of variable length (varying from 95 to 135 residues) and a very short C-terminus. It is the *L*. *infantum* orthologue of a microtubule-associated protein, described from *Trypanosoma brucei* as GB4 [32]. Lci8 consists of 61 identical repeats of 10 amino acid residues, flanked by short N and C-terminal regions of 331 and 242 residues in length, respectively. It is an orthologue of a *T*. *brucei* membrane associated protein possibly involved with vesicular transport, Tb-291 [33]. Based upon the gene sequence of its *L*. *major* orthologue, the full-length Lci9 is shorter than the other antigens discussed here and consists of two sets of related repeats of 25 (14 copies) and 34 (11 copies) residues, flanked by very short N and C-terminal regions (169 and 97 residues long, respectively). This is the orthologue of the protein named nucleoporin (TbNup140) [34]. Lci10 encodes a hypothetical protein that has orthologues in other *Leishmania* species but is absent from *Trypanosoma*, although it might be related to a protein found within the flagellar attachment zone in *T*. *brucei*. The sequence available from the *L*. *infantum* genome appears to be incomplete but the Lci10 clone contains multiple related repetitive motifs of different sizes (varying from 68 to 198 residues) which follow a non-repetitive N-terminal region. Lci12, also defined as a hypothetical protein, is the *Leishmania* orthologue of the membrane-associated protein Tb-292 from *T*. *brucei*, related to the Lci8 orthologue Tb-291 [33]. The *L*. *infantum* protein was also identified in a bioinformatic screening for proteins with tandem repeat domains [35]. It is composed by an N-terminal region containing approximately 160 amino acids, followed by a region containing 30 repeats of an 8 amino acids-long motif and a carboxi-terminal region containing the *trans*-membrane segments.

### Recombinant antigen expression

**Fig 2:**
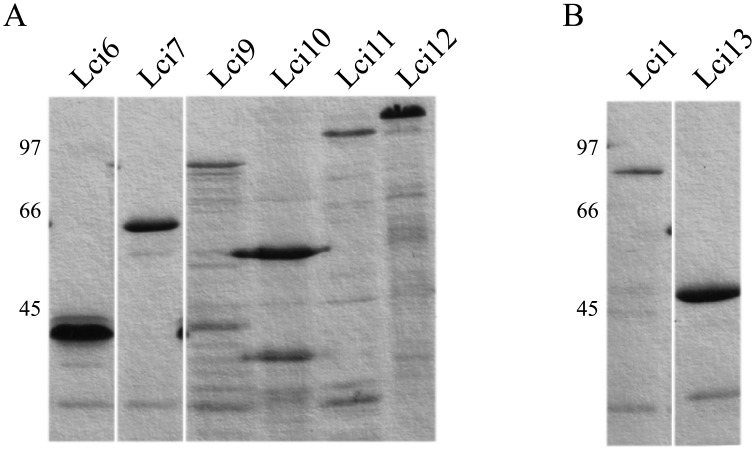
Polyacrilamide gel electrophoresis showing the affinity purified polypeptides evaluated in this study. (A) His-tagged, recombinant fragments of Lci6, Lci7, Lci9, Lci10, Lci11 and Lci12 in denaturing 15% SDS-PAGE stained with Coomassie Blue. All lanes shown are from a single gel but selected regions were removed for clarity. For some of these polypeptides, especially the larger ones, bands of lower molecular weight can be generally seen, but these are likely degradation products due to proteolysis within the bacteria that vary in intensity between different batches of purification. (B) The same for Lci1 and Lci13. The numbers on the left indicate the sizes of molecular weight markers.

With the exception of Lci8, all the other antigens identified here were efficiently expressed in *E*. *coli* and purified by affinity chromatography on nickel columns. Results of representative analysis by SDS-PAGE of the various recombinant proteins are shown in Fig 2a. For Lci6, based on the subcloned fragment, the recombinant protein would encompass residues 246 to 1548 of the original polypeptide with a predicted molecular weight of 286 kDa. However, a single band of ~40 kDa (a likely result of internal proteolytic cleavage) is seen after expression and purification; it includes the His-tag at the N-terminus and approximately the first one-third of the protein. The Lci7 subcloned fragment encodes for a polypeptide consisting of most of the protein (residues 69 to 547), generating a 54 kDa recombinant protein. For Lci9, the recombinant polypeptide consists of 799 residues and includes all elements identified within its *L*. *major* orthologue, expressed as a 71 kDa band. Lci10 was only expressed as a polypeptide encompassing residues 404 to 717 of the full-length protein and a predicted molecular weight of ~35 kDa (Fig 1d). The recombinant protein, nevertheless, migrates with an apparent molecular weight greater than 50 kDa. Recombinant Lci11 encompasses residues 47 to 688 of the original protein with a predicted molecular weight of 63 kDa, but also migrates in gel with an apparent molecular weight higher than predicted, ~100 kDa. For Lci12, the recombinant polypeptide expressed comprises residues 153 to 1081 of the full-length protein, with a predicted size of ~106 kDa and a compatible migration in gel.

Two other proteins obtained in previous works were added to this study for comparative purposes and shown in Fig 2b. Lci1 encodes a *L*. *infantum* homologue of the cytoplasmic heat shock protein HSP70 [23] and the recombinant protein migrates with an apparent molecular weight of ~80 kDa. The second protein, named Lci13 here for clarity, encodes part of the *L*. *infantum* mitochondrial HSP70. The recombinant fragment evaluated was described before [28] and migrates in gel with an apparent molecular weight of ~45 kDa. Despite the fact that both Lci1 and Lci13 belong to the family of HSP70 proteins, the identity between the two in terms of amino acid sequence is less than 50% and a rabbit polyclonal serum produced against recombinant Lci13 does not recognize Lci1 (unpublished data).

### Recognition of the *L*. *infantum* recombinant proteins by human sera

**Fig 3:**
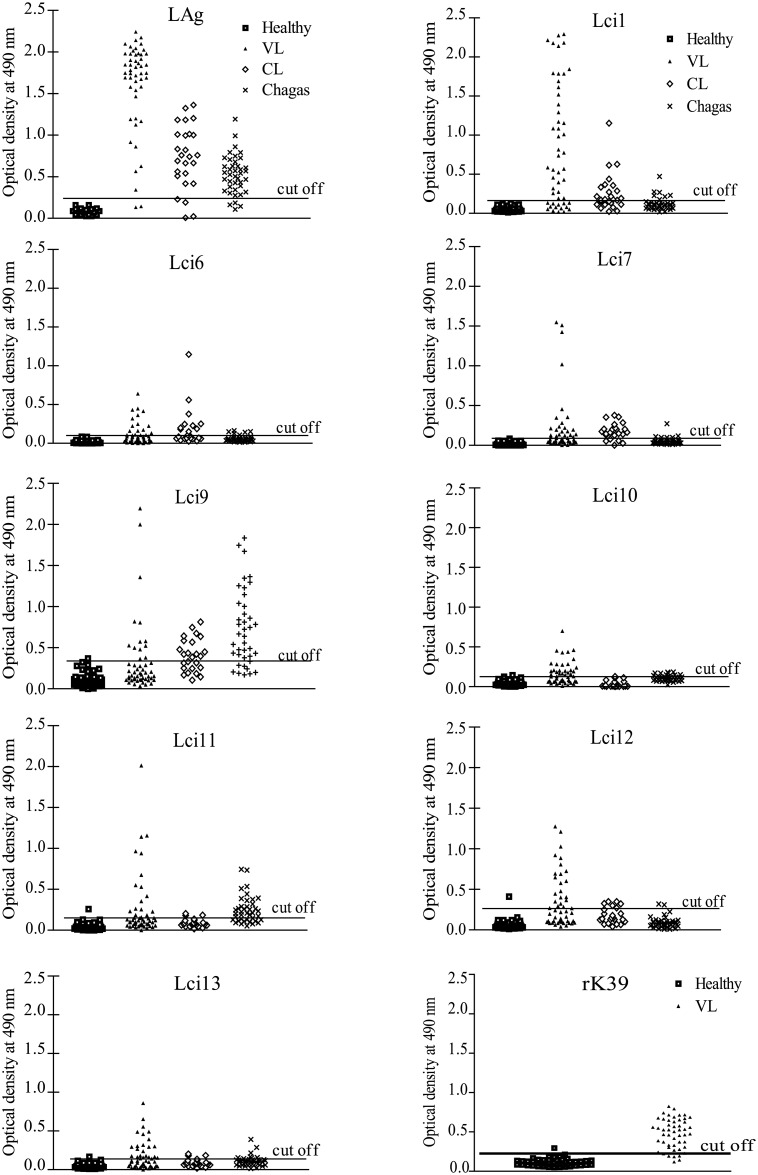
Evaluation of the novel *Leishmania infantum* recombinant antigens for the diagnosis of visceral leishmaniasis in humans. A panel of human sera derived from individuals with confirmed visceral leishmaniasis was tested through an Enzyme-Linked ImmunoSorbent Assay (ELISA) with the recombinant antigens produced in this study, the rK39 and the total *L*. *infantum* lysate (LAg). The panel was composed of 50 serum samples from individuals with visceral leishmaniasis (VL), 50 serum samples from healthy individuals (Healthy), 23 serum samples from individuals with cutaneous leishmaniasis (CL) and 40 serum samples from individuals with Chagas disease (Chagas). Each symbol corresponds to the result obtained with an individual serum. The horizontal lines indicate cutoff values, calculated by the means of results obtained with serum samples from healthy donors plus three standard deviations.

To evaluate the antigenicity of the recombinant antigens selected for this study, we performed ELISA assays with serum from humans infected with *L*. *infantum* and with VL diagnosis confirmed through parasitological tests (Fig 3 and Table 2). With the exception of Lci1, previously tested [23], none of the others recombinant polypeptides had been evaluated before in similar assays. The different antigens produced ELISA reactions with variable intensities but the sensitivity values for the novel antigens were low, varying between 26 and 48%, and much inferior to the performance seen with Lci1 (72%), also insufficient, or with either the total parasite lysate (LAg– 96%) or the commercial recombinant rK39 antigen (84%). As before [23], and in order to minimize the possibility of false positive results and increase specificity, for these experiments we opted to define a cutoff based on the mean plus three standard deviations of the results generated with control sera from healthy individuals. Indeed, most of the antigens did not produce false positive reactions and the specificity values calculated based on these sera were equal to or very close to 100% (the data also summarized in Fig 3 and Table 2). The various antigens were also tested with sera from patients with cutaneous leishmaniasis and Chagas’ disease in order to evaluate their cross-reactivity for VL diagnosis. Remarkably, Lci9 produced very strong cross-reactions with sera from Chagas’ disease patients and only Lci10 and Lci12 did not display cross-reactions to sera from either cutaneous leishmaniasis or Chagas’ disease individuals. With the exception of Lci9 (60%), the specificity values calculated based on these sera were all above 95%. Overall, among the new recombinant polypeptides tested, antigens Lci1 and Lci7 produced a better performance (greater sensitivity with very high specificity), but their low sensitivity compromises their use as single antigens for the diagnosis of human VL (Table 2).

**Table 2:** Summary of the ELISA assays carried out with human sera in order to evaluate the performance of the recombinant antigens for the identification of positive cases of human VL. LAg represents the total *L*. *infantum* lysate used as positive control. C.I. stands for confidence interval. NE—Not Evaluated.

	Sera from confirmed VL infected humans		Sera from related diseases (% of cross-reactive sera with positive results)		
Recombinant antigens	% of sensitivity (99% C.I.)	% of specificity/ healthy sera (99% C.I.)	Cutaneous Leishmaniasis	Chagas’ disease	% of specificity/ related diseases (99% C.I.)
**LAg**	96% (86.3%–99.5%)	100% (92.9%–100%)	52%	20%	74% (62.7%–82.6%)
**Lci1**	72% (57.5%–83.8%)	100% (92.9%–100%)	26%	2.5%	94% (87.65%–97.47%)
**Lci6**	48% (33.7%–62.6%)	100% (92.9%–100%)	13%	0%	97% (92.4%–99.4%)
**Lci7**	52% (37.4%–66.3%)	100% (92.9%–100%)	17%	2.5%	96% (89.98%–98.55%)
**Lci9**	26% (14.6%–40.3%)	98% (89.2%–100%)	56%	77.5%	60% (50.4%–69%)
**Lci10**	48% (33.7%–62.6%)	100% (92.89%–100%)	0%	0%	100% (92.9%–100%)
**Lci11**	32% (19.5% -46.7%)	98% (89.2%–100%)	25%	7.5%	90% (82.5%–94.5%)
**Lci12**	46% (31.8% -60.7%)	100% (92.9%–100%)	0%	0%	99% (95.2%–100%)
**Lci13**	44% (30% -58.8%)	98% (89.4%–100%)	12.5%	12.5%	93% (86.5%–96.9%)
**Mix (Lci1, Lci12 and Lci13)**	84% (70.9%–92.8%)	98% (86.8%–99.9)	NE	NE	NE
**rK39**	84% (70.9%–92.8%)	100% (92.89%–100%)	NE	NE	NE

### Recognition of the *L*. *infantum* recombinant proteins by canine sera

**Fig 4:**
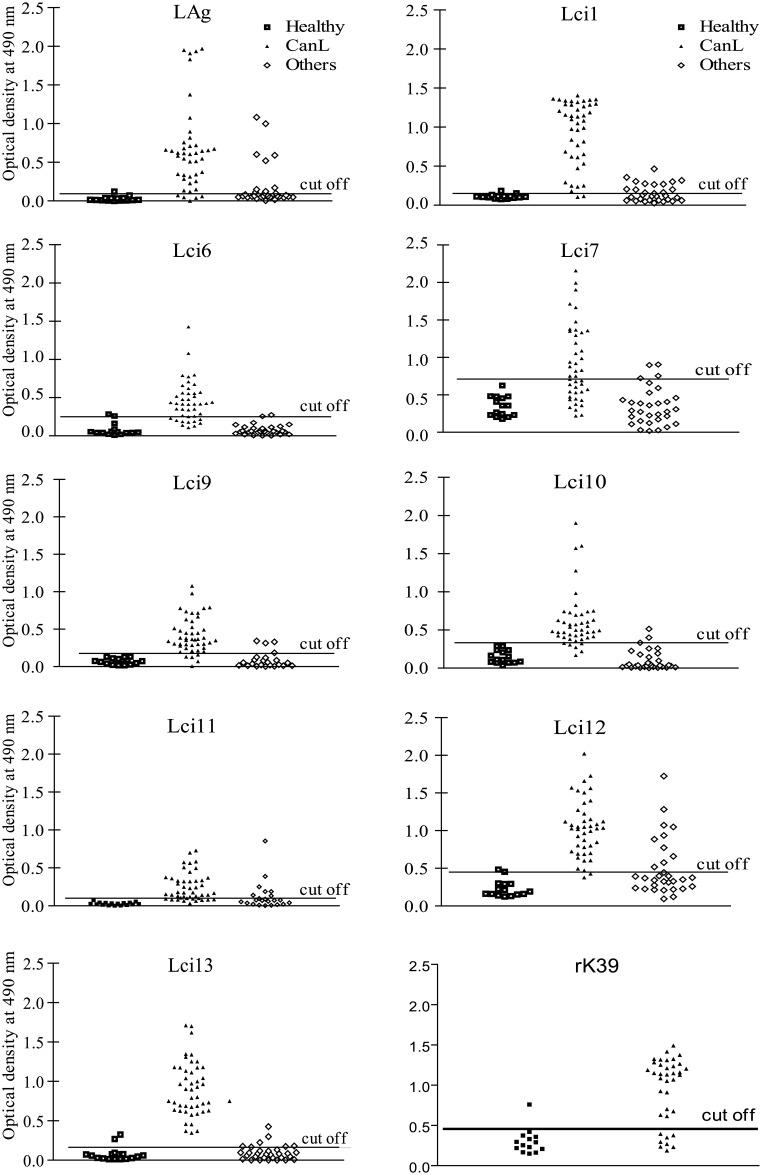
Evaluation of the novel *Leishmania infantum* recombinant antigens for the diagnosis of canine visceral leishmaniasis. The ELISA assay described in the previous Figure was performed also with a panel of canine sera from animals confirmed with visceral leishmaniasis. Serum samples from 46 dogs with leishmaniasis confirmed through parasitological tests (CanL—canine leishmaniasis), 31 dogs with other infections (4 with babesiosis, 20 with erhlichiosis, and 7 with demodicosis—Others) and 15 healthy control animals (Healthy) were assayed with the recombinant antigens, the rK39 and the total *L*. *infantum* lysate (LAg). Each symbol corresponds to the result obtained with an individual serum. The horizontal lines indicate cutoff values, calculated as described in Materials and Methods.

Next, we assayed the same set of antigens for their potential to identify positive sera from dogs with parasitologically confirmed leishmaniasis. Again, only Lci1 had been previously been tested in similar assays [23]. In general, these antigens had a performance with the canine sera better than that seen for the positive human samples. The ELISA’s sensitivity varied between 49 and 91%, for the novel antigens described here, and 93 and 97%, for Lci1 and Lci13, respectively, comparable to that from the total parasite lysate (LAg– 93%). In contrast, the sensitivity for the commercial rK39 was 68%. Again no false positive results were seen when sera from healthy control dogs were evaluated, with the specificity values calculated based on these equal to 100% for the different polypeptides tested. When sera from dogs afflicted with other infectious diseases (erhlichiosis, babesiosis or demodicosis) were evaluated, however, some positive cross-reactions, with moderate intensity were observed, leading to reduced specificity values calculated with these sera (76%–100%) (Fig 4 and Table 3). Lci13 displayed the best performance between the various antigens tested, with 97% sensitivity and very high specificity. Its performance was even superior to the one seen with the assays using total parasite lysate (Lag), which displayed some false negative results as well as strong cross-reactions with the sera from dogs with other infectious diseases. Other antigens also produced strong reactions with the positive dogs’ sera (Lci1 and the novel antigen Lci12), but their performance was inferior to Lci13. Nevertheless, with the dog sera at least, all three antigens performed much better than the commercial recombinant rK39 and can be potentially useful as part of novel tests for the diagnosis of canine leishmaniasis.

**Table 3:** Performance of the recombinant antigens with dog sera. LAg represents the total *L*. *infantum* lysate used as positive control. C.I. stands for confidence interval. NE—Not Evaluated.

	Sera from dogs with confirmed leishmaniasis		Sera from related diseases (% of cross-reactive sera with positive results)			
Recombinant antigens	% of sensitivity (99% C.I.)	% of specificity/ healthy sera (99% C.I.)	Ehrlichia	Babesiosis	Demodicosis	% of specificity/ related diseases (99% C.I.)
**LAg**	93% (80.9%–98.5%)	100% (76.8%–100%)	15%	50%	0%	89% (76.4%–96.4%)
**Lci1**	93% (80.9%–98.5%)	100% (76.8%–100%)	50%	0%	14%	76% (61.2%–87.4%)
**Lci6**	67% (51.5%–80.9%)	100% (76.8%–100%)	0%	0%	0%	100% (76.8%–100%)
**Lci7**	49% (33.3%–64.6%)	100% (76.8%–100%)	15%	0%	0%	94% (82.1%–98.6%)
**Lci9**	85% (71.1%–93.7%)	100% (76.8%–100%)	10%	0%	14%	92% (78.6%–98.3%)
**Lci10**	77% (61.4%–88.2%)	100% (76.8%–100%)	15%	0%	14%	91% (79.2%–97.6%)
**Lci11**	83% (68.6%–92.2%)	100% (76.8%–100%)	30%	0%	28%	79% (62.7%–90.4%)
**Lci12**	91% (77.9% -97.4%)	100% (76.8%–100%)	35%	50%	0%	80% (66.1%–90.6%)
**Lci13**	97% (85.8%–99.9%)	100% (76.8%–100%)	5%	0%	0%	98% (88.5%–99.9%)
**Mix (Lci1, Lci12 and Lci13)**	88% (72.6%–96.7%)	100% (76.8%–100%)	NE	NE	NE	NE
**rK39**	68% (50.2%–82%)	100% (78.2%–100%)	NE	NE	NE	NE

### Evaluation of a mix of three recombinant proteins against both human and canine sera

**Fig 5:**
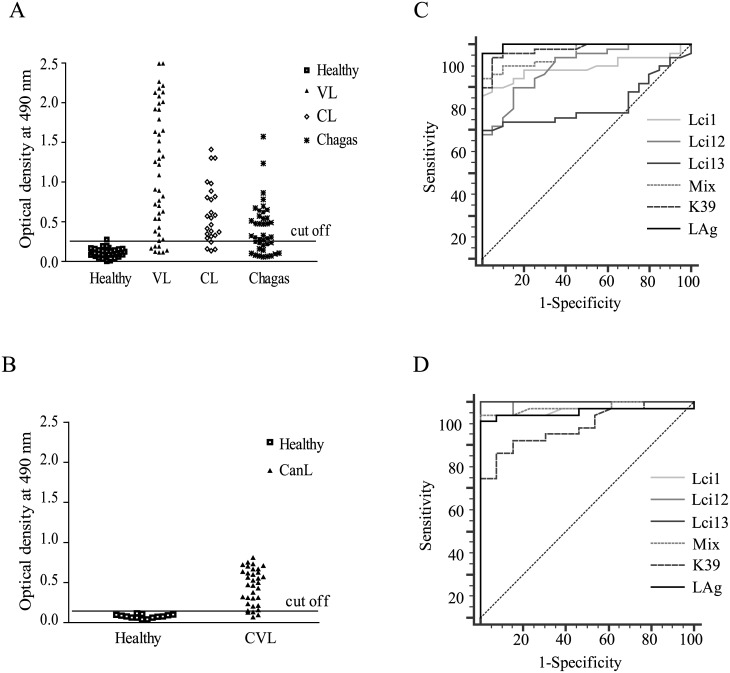
Reactivity of an antigenic mix with human and canine sera positive for visceral leishmaniasis. (A) Reactivity of an antigenic mix with human sera from individuals with confirmed visceral leishmaniasis (VL), cutaneous leishmaniasis (CL), Chagas’ disease (Chagas) and healthy controls (Healthy). (B) ROC curves displaying the performance with the human sera of the protein “Mix”, the individual recombinant proteins that were included in the “Mix”, rK39 and the total *L*. *infantum* lysate (LAg). (C) Serum reactivity of dogs with canine leishmaniasis (CanL) and healthy control animals (Healthy) with the same protein “Mix”. (D) SROC curve showing the performance with the canine sera of the protein “Mix”, the individual recombinant proteins that were included in the “Mix”, rK39 and the total *L*. *infantum* lysate.

When the results from the ELISA assays with the VL positive human sera were analyzed in more detail, all were seen to recognize at least one of the recombinant antigens evaluated (data not shown). This led us to test in similar assays, with the VL positive sera from humans and dogs, a mix of recombinant proteins with complementary reactivities (Lci1, Lci12, Lci13). This “Mix” consists of the best three proteins evaluated here in terms of sensitivity and specificity with human and/or canine sera. The results from the assays with the human sera are shown in Fig 5a. The “Mix” produced a strong reaction with the positive sera, displaying a greater sensitivity when compared with the individual antigens (84% sensitivity—also shown in Table 2), and an overall performance more similar to the total parasite lysate or to recombinant rK39 when the results were analyzed through a ROC curve (Fig 5b). A great number of cross-reactive reactions, however, were seen with the sera from Chagas’ disease and cutaneous leishmaniasis individuals. The assays using the canine sera also resulted in a significant proportion of positive results for the animals with confirmed leishmaniasis (88% sensitivity—Fig 5c and Table 2). The “Mix”, however, produced a lower performance than the one observed for Lci13 and the total parasite lysate, although no significant difference was seen when these results were analyzed through a ROC curve (Fig 5d), with all three samples showing strong sensitivity and specificity for the diagnosis of the canine leishmaniasis. In comparison with rK39, however, the “Mix” still behaved much better than the commercially produced recombinant protein when tested with the dog sera.

## Discussion

An early and accurate diagnosis of VL is of great importance to the administration of an effective treatment, screening of endemic areas and consequent interruption of the parasite life cycle [15,16]. In this study, a panel of novel *L*. *infantum* antigens was evaluated for their potential use for the diagnosis of both human and canine forms of VL. ELISA was chosen for this evaluation since it is a preferred choice for the serodiagnosis of the disease in the laboratory [36] and it has been shown to be more reliable and sensitive than other rapid tests [37]. Several of the antigens tested showed very good potential for the canine form, with one of them (Lci13), demonstrating a better capacity to detect the canine leishmaniasis than the total parasite lysate. This protein is a promising antigen, since some recent studies did not find a single protein capable of such diagnostic efficiency in dogs [38,39]. In contrast, none of the antigens tested were as effective for human VL, highlighting the differences in immune response between the two different disease targets, as highlighted previously [23,26]. In the assays described here, the commercial rK39 antigen was included in order to facilitate the comparison of the newly identified antigens with other previously described and also to evaluate the immune response level from the sera selected for this study. The results for the rK39 were indeed consistent with what has been reported in the literature and confirming that it is effective for the diagnosis of human VL but performs much less satisfactorily with the canine form of the disease [19,24].

Most of the antigens tested here were proteins bearing repetitive motifs. Such proteins have been reported in the literature to be present in various organisms, from viruses to humans, and are characterized by the presence of at least two or more copies of an amino acid sequence. Several studies show that they are particularly antigenic and it is believed that this occurs due to stimulation of B cells by binding these repetitive antigens, by a route independent of the T-lymphocyte stimulation [40–43]. For these reasons, these proteins are strong candidates for the development of serological assays and vaccine targets. Here, the evaluated proteins bearing repetitive motifs did not show good sensitivity in humans. However, when tested against dog sera, they displayed high sensitivity, corroborating previously published data where a repetitive protein showed higher sensitivity than non-repetitive proteins with canine samples [44].

The tested recombinant antigens displayed major differences in sensitivity when tested in human and dog sera, as previously seen by us and others using different recombinant polypeptides in the same type of assay [23,26]. This variance may be due to the differentiated way that the vertebrate hosts react to the parasite, mainly regarding the recognition and presentation of the distinct antigens studied to the immune system or, as has been proposed [26], this response may be due to different mechanisms for parasite survival in each host (man and dog). On the other hand, since it has been shown that variations in sensitivity may be due to the symptomatic and asymptomatic phases of the disease, the symptomatic phase displaying the best sensitivity performance in the serological tests [45], these differences may, to some extent, reflect the stage of the disease in the individuals from whom the sera were collected. In general, the differential recognition of the studied proteins by the sera from the two hosts highlights the differences in the immune responses elicited by the parasite and the need to optimize the current serological tests.

In order to improve the diagnosis of VL in humans, we proposed the evaluation of an antigen “Mix”, composed by antigens which already had produced good performance with the dog samples. These antigens were chosen in order to combine the high sensitivity and specificity found in dog sera and improve the tests made with the individual proteins to detect the disease in dog and humans, resulting in a promising serological test. Recombinant Lci1 had been evaluated before with a good performance for VL immunodiagnosis in dogs [23] and it was selected as one of the three proteins (with Lci12 and Lci13) included in the “Mix”. The use of this “Mix” led to a substantial increase in the sensitivity for the human disease, with a minor decrease in performance for the canine leishmaniasis, when compared with Lci13 alone and total *Leishmania* lysate, with no false positive results seen when assayed against the healthy control sera. The “Mix” was therefore a significant improvement as a tool to detect the disease in both humans and dogs, although further optimization would still be required in antigen representation in other to increase sensitivity prior to any considerations regarding a commercial test. Nevertheless, these results contrast with a recent report where a similar mix of three antigens did not lead to an increase in their diagnostic performance when it was compared with the individual proteins alone [39]. Lack of significant improvement attempted by a mixture of proteins may be explained by the decrease of the antigenicity of each individual protein, due to the presence of the antigenic peptide in lower concentration in the solid phase. An alternative serological method that could be used to deal with the limitations presented by the protein mixture is the development of chimeric proteins, containing the regions of the proteins that presented the best performance in serological evaluations. Some studies in this area have already been done and they showed a significant improvement in the sensitivity of the serological test [46–49].

In summary, the recombinant antigens tested individually in this study in human and dog sera, displayed different sensitivities for the serodiagnosis of VL, with a better performance in dogs. In fact, Lci13 displayed a sensitivity for the dog sera higher than the current field tests, which demonstrates the potential for this recombinant antigen to detect CVL on its own. The goal of identifying a single natural antigen valid for the diagnosis of both forms of the disease does not seem viable. Recently, the use of chimeric proteins based on multiple antigenic peptides for the diagnosis of the canine leishmaniasis has been tried with promising results. The performance seen with the protein mix tested here indicates that, with improvement, the inclusion of selected epitopes from these antigens into novel chimeric proteins could be a good option to obtain serological tests with higher sensitivity and which would simultaneously be effective for both species.

## References

[1] Guerin PJ, Olliaro P, Sundar S, Boelaert M, Croft SL, Desjeux P, et al Visceral leishmaniasis: current status of control, diagnosis, and treatment, and a proposed research and development agenda. Lancet InfectDis. 2002;2: 494–501. doi: 10.1016/S1473-3099(02)00347-X 10.1016/s1473-3099(02)00347-x 12150849

[2] Harhay MO, Olliaro PL, Vaillant M, Chappuis F, Lima MA, Ritmeijer K, et al Who is a typical patient with visceral leishmaniasis? Characterizing the demographic and nutritional profile of patients in Brazil, East Africa, and South Asia. AmJTropMedHyg. 2011;84: 543–550. 10.4269/ajtmh.2011.10-0321 PMC3062446 21460007

[3] Shakya N, Bajpai P, Gupta S. Therapeutic switching in leishmania chemotherapy: a distinct approach towards unsatisfied treatment needs. JParasitDis. 2011;35: 104–112. 10.1007/s12639-011-0040-9 PMC3235389 23024489

[4] Singh N, Kumar M, Singh RK. Leishmaniasis: current status of available drugs and new potential drug targets. Asian PacJTropMed. 2012;5: 485–497. 10.1016/S1995-7645(12)60084-4 22575984

[5] Desjeux P. The increase in risk factors for leishmaniasis worldwide. TransRSocTropMedHyg. 2001;95: 239–243. 10.1016/s0035-9203(01)90223-8 11490989

[6] Maia-Elkhoury ANS, Alves WA, De Sousa-Gomes ML, De Sena JM, Luna E a. Visceral leishmaniasis in Brazil: trends and challenges. CadSaude Publica. 2008;24: 2941–2947. doi: 10.1590/S0102-311X2008001200024 10.1590/s0102-311x2008001200024 19082286

[7] Martins-Melo FR, Lima MS, Alencar CH, Ramos AN Jr., Heukelbach J. Epidemiological patterns of mortality due to visceral leishmaniasis and HIV/AIDS co-infection in Brazil, 2000–2011. TransRSocTropMedHyg. 2014;108: 338–347. 10.1093/trstmh/tru050 24706340

[8] Quinnell RJ, Courtenay O. Transmission, reservoir hosts and control of zoonotic visceral leishmaniasis. Parasitology. 2009;136: 1915–1934. doi: 10.1017/S0031182009991156 19835643 10.1017/S0031182009991156

[9] Roque ALR, Jansen AM. Wild and synanthropic reservoirs of *Leishmania* species in the Americas. IntJParasitolParasitesWildl. 2014;3: 251–262. doi: 10.1016/j.ijppaw.2014.08.004 25426421 10.1016/j.ijppaw.2014.08.004 PMC4241529

[10] Noli C, Saridomichelakis MN. An update on the diagnosis and treatment of canine leishmaniosis caused by *Leishmania infantum* (syn. L. chagasi). Vet J. Elsevier Ltd; 2014;202: 425–35. doi: 10.1016/j.tvjl.2014.09.002 25266647 10.1016/j.tvjl.2014.09.002

[11] Gomes YM, Paiva CM, Lira RA, Abath FG, Alves LC. Diagnosis of canine visceral leishmaniasis: biotechnological advances. VetJ. 2008;175: 45–52. 17150389 10.1016/j.tvjl.2006.10.019

[12] Maia C, Campino L. Methods for diagnosis of canine leishmaniasis and immune response to infection. VetParasitol. 2008;158: 274–287. 10.1016/j.vetpar.2008.07.028 18789583

[13] Romero GA, Boelaert M. Control of visceral leishmaniasis in latin america-a systematic review. PLoSNeglTropDis. 2010;4: e584. 10.1371/journal.pntd.0000584 PMC2808217 20098726

[14] Srivastava P, Dayama A, Mehrotra S, Sundar S. Diagnosis of visceral leishmaniasis. TransRSocTropMedHyg. 2011;105: 1–6. 10.1016/j.trstmh.2010.09.006 PMC2999003 21074233

[15] Srividya G, Kulshrestha A, Singh R, Salotra P. Diagnosis of visceral leishmaniasis: developments over the last decade. ParasitolRes. 2012;110: 1065–1078. 10.1007/s00436-011-2680-1 22065060

[16] Singh OP, Sundar S. Developments in diagnosis of visceral leishmaniasis in the elimination era. Journal of Parasitology Research. 2015 doi: 10.1155/2015/239469 26843964 10.1155/2015/239469 PMC4710934

[17] Burns JM Jr., Shreffler WG, Benson DR, Ghalib HW, Badaro R, Reed SG, et al Molecular characterization of a kinesin-related antigen of *Leishmania* chagasi that detects specific antibody in African and American visceral leishmaniasis. ProcNatlAcadSciUSA. 1993;90: 775–779. doi: 10.1073/pnas.90.2.775 10.1073/pnas.90.2.775 PMC45748 8421715

[18] Singh S, Gilman-Sachs A, Chang KP, Reed SG. Diagnostic and prognostic value of K39 recombinant antigen in Indian leishmaniasis. JParasitol. 1995;81: 1000–1003. 8544037

[19] Maia Z, Lirio M, Mistro SS, Mendes CMCMC, Mehta SR, Badaro R, et al Comparative study of rK39 *Leishmania* antigen for serodiagnosis of visceral leishmaniasis: Systematic review with meta-analysis. PLoS Negl Trop Dis. 2012;6: e1484 doi: 10.1371/journal.pntd.0001484 22303488 10.1371/journal.pntd.0001484 PMC3269412

[20] Houghton RL, Petrescu M, Benson DR, Skeiky YA, Scalone A, Badaro R, et al A cloned antigen (recombinant K39) of *Leishmania* chagasi diagnostic for visceral leishmaniasis in human immunodeficiency virus type 1 patients and a prognostic indicator for monitoring patients undergoing drug therapy. JInfectDis. 1998;177: 1339–1344. 10.1086/515289 9593022

[21] Boelaert M, El-Safi S, Hailu A, Mukhtar M, Rijal S, Sundar S, et al Diagnostic tests for kala-azar: a multi-centre study of the freeze-dried DAT, rK39 strip test and KAtex in East Africa and the Indian subcontinent. TransRSocTropMedHyg. 2008;102: 32–40. 10.1016/j.trstmh.2007.09.003 17942129

[22] Singh S, Kumari V, Singh N. Predicting kala-azar disease manifestations in asymptomatic patients with latent *Leishmania* donovani infection by detection of antibody against recombinant K39 antigen. ClinDiagnLab Immunol. 2002;9: 568–572. 10.1128/CDLI.9.3.568-572.2002 PMC119988 11986261

[23] Oliveira GG, Magalhaes FB, Teixeira MC, Pereira AM, Pinheiro CG, Santos LR, et al Characterization of novel *Leishmania infantum* recombinant proteins encoded by genes from five families with distinct capacities for serodiagnosis of canine and human visceral leishmaniasis. AmJTropMedHyg. 2011;85: 1025–1034. 10.4269/ajtmh.2011.11-0102 PMC3225146 22144438

[24] Quinnell RJ, Carson C, Reithinger R, Garcez LM, Courtenay O. Evaluation of rK39 rapid diagnostic tests for canine visceral leishmaniasis: longitudinal study and meta-analysis. PLoSNeglTropDis. 2013;7: e1992. 10.1371/journal.pntd.0001992 PMC3542187 23326615

[25] Peixoto HM, de Oliveira MR, Romero GA. Serological diagnosis of canine visceral leishmaniasis in Brazil: systematic review and meta-analysis. TropMedIntHealth. 2015;20: 334–352. 10.1111/tmi.12429 25403359

[26] Goto Y, Howard RF, Bhatia A, Trigo J, Nakatani M, Netto EM, et al Distinct antigen recognition pattern during zoonotic visceral leishmaniasis in humans and dogs. VetParasitol. 2009;160: 215–220. doi: 10.1016/j.vetpar.2008.10.097 19059724 10.1016/j.vetpar.2008.10.097 PMC2947490

[27] Teixeira MC, Oliveira GG, Silvany MA, cantara-Neves NM, Soares MB, Ribeiro-Dos-Santos R, et al A strategy for identifying serodiagnostically relevant antigens of *Leishmania* or other pathogens in genetic libraries. Biologicals. 2007;35: 51–54. doi: 10.1016/j.biologicals.2006.01.005 16580229 10.1016/j.biologicals.2006.01.005

[28] Campos RM, Nascimento M, Ferraz JC, Pereira MM, Rocha PO, Thompson GM, et al Distinct mitochondrial HSP70 homologues conserved in various *Leishmania* species suggest novel biological functions. MolBiochemParasitol. 2008;160: 157–162. 10.1016/j.molbiopara.2008.04.013 18541316

[29] Zinoviev A, Leger M, Wagner G, Shapira M. A novel 4E-interacting protein in *Leishmania* is involved in stage-specific translation pathways. Nucleic Acids Res. 2011;39: 8404–8415. doi: 10.1093/nar/gkr555 21764780 10.1093/nar/gkr555 PMC3201875

[30] Webb JR, Kaufmann D, Campos-Neto A, Reed SG. Molecular cloning of a novel protein antigen of *Leishmania* major that elicits a potent immune response in experimental murine leishmaniasis. JImmunol. 1996;157: 5034–5041. 8943412

[31] Peacock CS, Seeger K, Harris D, Murphy L, Ruiz JC, Quail MA, et al Comparative genomic analysis of three *Leishmania* species that cause diverse human disease. NatGenet. 2007;39: 839–847. 10.1038/ng2053 PMC2592530 17572675

[32] Rindisbacher L, Hemphill A, Seebeck T. A repetitive protein from *Trypanosoma brucei* which caps the microtubules at the posterior end of the cytoskeleton. MolBiochemParasitol. 1993;58: 83–96. doi: 10.1016/0166-6851(93)90093-D 10.1016/0166-6851(93)90093-d 8459837

[33] Lee MG, Russell DG, D’Alesandro PA, Van der Ploeg LH. Identification of membrane-associated proteins in *Trypanosoma brucei* encoding an internal, EARLRAEE amino acid repeat. JBiolChem. 1994;269: 8408–8415. 8132566

[34] DeGrasse JA, DuBois KN, Devos D, Siegel TN, Sali A, Field MC, et al Evidence for a shared nuclear pore complex architecture that is conserved from the last common eukaryotic ancestor. MolCell Proteomics. 2009;8: 2119–2130. 10.1074/mcp.M900038-MCP200 PMC2742445 19525551

[35] Goto Y, Coler RN, Reed SG. Bioinformatic identification of tandem repeat antigens of the *Leishmania donovani* complex. InfectImmun. 2007;75: 846–851. 10.1128/IAI.01205-06 PMC1828517 17088350

[36] Elmahallawy EK, Sampedro MA, Rodriguez-Granger J, Hoyos-Mallecot Y, Agil A, Navarro Mari JM, et al Diagnosis of leishmaniasis. JInfectDevCtries. 2014;8: 961–972. doi: 10.3855/jidc.4310 25116660 10.3855/jidc.4310

[37] Abass E, Kang C, Martinkovic F, Semiao-Santos SJ, Sundar S, Walden P, et al Heterogeneity of *Leishmania donovani* parasites complicates diagnosis of visceral leishmaniasis: comparison of different serological tests in three endemic regions. PLoSOne. 2015;10: e0116408. 10.1371/journal.pone.0116408 PMC4348478 25734336

[38] Fraga DB, Da Silva ED, Pacheco L V, Borja LS, de O I, Coura-Vital W, et al A multicentric evaluation of the recombinant *Leishmania infantum* antigen-based immunochromatographic assay for the serodiagnosis of canine visceral leishmaniasis. ParasitVectors. 2014;7: 136. 10.1186/1756-3305-7-136 PMC3972511 24684857

[39] Fonseca AM, Faria AR, Rodrigues FTG, Nagem RAP, Magalhães RDM, Cunha JLR, et al Evaluation of three recombinant *Leishmania infantum* antigens in human and canine visceral leishmaniasis diagnosis. Acta Trop. 2014;137: 25–30. doi: 10.1016/j.actatropica.2014.04.028 24801885 10.1016/j.actatropica.2014.04.028

[40] Vos Q, Lees A, Wu ZQ, Snapper CM, Mond JJ. B-cell activation by T-cell-independent type 2 antigens as an integral part of the humoral immune response to pathogenic microorganisms. ImmunolRev. 2000;176: 154–170. 10.1034/j.1600-065x.2000.00607.x 11043775

[41] Goto Y, Carter D, Reed SG. Immunological dominance of *Trypanosoma cruzi* tandem repeat proteins. InfectImmun. 2008;76: 3967–3974. 10.1128/IAI.00604-08 PMC2519453 18625739

[42] Goto Y, Carter D, Guderian J, Inoue N, Kawazu S, Reed SG. Upregulated expression of B-cell antigen family tandem repeat proteins by *Leishmania* amastigotes. InfectImmun. 2010;78: 2138–2145. 10.1128/IAI.01102-09 PMC2863543 20160013

[43] Valiente-Gabioud AA, Veaute C, Perrig M, Galan-Romano FS, Sferco SJ, Marcipar IS. Effect of repetitiveness on the immunogenicity and antigenicity of *Trypanosoma cruzi* FRA protein. ExpParasitol. 2011;127: 672–679. doi: 10.1016/j.exppara.2010.11.011 21118687 10.1016/j.exppara.2010.11.011

[44] Rosati S, Ortoffi M, Profiti M, Mannelli A, Mignone W, Bollo E, et al Prokaryotic expression and antigenic characterization of three recombinant *Leishmania* antigens for serological diagnosis of canine leishmaniasis. ClinDiagnLab Immunol. 2003;10: 1153–1156. 10.1128/CDLI.10.6.1153-1156.2003 PMC262443 14607883

[45] Mettler M, Grimm F, Capelli G, Camp H, Deplazes P. Evaluation of enzyme-linked immunosorbent assays, an immunofluorescent-antibody test, and two rapid tests (immunochromatographic-dipstick and gel tests) for serological diagnosis of symptomatic and asymptomatic *Leishmania* infections in dogs. JClinMicrobiol. 2005;43: 5515–5519. 10.1128/JCM.43.11.5515-5519.2005 PMC1287801 16272479

[46] Boarino A, Scalone A, Gradoni L, Ferroglio E, Vitale F, Zanatta R, et al Development of recombinant chimeric antigen expressing immunodominant B epitopes of *Leishmania infantum* for serodiagnosis of visceral leishmaniasis. ClinDiagnLabImmunol. 2005;12: 647–653. doi: 10.1128/CDLI.12.5.647-653.2005 10.1128/CDLI.12.5.647-653.2005 PMC1112073 15879027

[47] Camussone C, Gonzalez V, Belluzo MS, Pujato N, Ribone ME, Lagier CM, et al Comparison of recombinant *Trypanosoma cruzi* peptide mixtures versus multiepitope chimeric proteins as sensitizing antigens for immunodiagnosis. ClinVaccineImmunol. 2009;16: 899–905. 10.1128/CVI.00005-09 PMC2691048 19339486

[48] Castro-Junior JG, Freire ML, Campos SP, Scopel KK, Porrozzi R, Da Silva ED, et al Evidence of *Leishmania* (*Leishmania*) *infantum* infection in dogs from Juiz de Fora, Minas Gerais State, Brazil, based on immunochromatographic dual-path platform (DPP(R)) and PCR assays. RevInstMedTrop Sao Paulo 2014;56: 225–229. 10.1590/S0036-46652014000300008 PMC4085865 24879001

[49] Faria AR, de Castro Veloso L, Coura-Vital W, Reis AB, Damasceno LM, Gazzinelli RT, et al Novel Recombinant Multiepitope Proteins for the Diagnosis of Asymptomatic Leishmania infantum-Infected Dogs. PLoS Negl Trop Dis. 2015;9: 13–16. doi: 10.1371/journal.pntd.0003429 25569685 10.1371/journal.pntd.0003429 PMC4287523

